# Combination treatment for myeloproliferative neoplasms using JAK and pan-class I PI3K inhibitors

**DOI:** 10.1111/jcmm.12156

**Published:** 2013-11-19

**Authors:** Meng Ling Choong, Christian Pecquet, Vishal Pendharkar, Carmen C Diaconu, Jacklyn Wei Yan Yong, Shi Jing Tai, Si Fang Wang, Jean-Philippe Defour, Kanda Sangthongpitag, Jean-Luc Villeval, William Vainchenker, Stefan N Constantinescu, May Ann Lee

**Affiliations:** a Experimental Therapeutics Centre, Agency for Science Technology and ResearchSingapore; b Ludwig Institute for Cancer Research & de Duve Institute, UniversitéCatholique de LouvainBrussels, Belgium; c Stefan S. Nicolau Institute of VirologyBucharest, Romania; d INSERM U1009 InstitutGustaveRoussy, Université Paris-SudVillejuif Cedex, France

**Keywords:** combination treatment, kinases, myeloproliferative neoplasms, JAK2, PI3K

## Abstract

Current JAK2 inhibitors used for myeloproliferative neoplasms (MPN) treatment are not specific enough to selectively suppress aberrant JAK2 signalling and preserve physiological JAK2 signalling. We tested whether combining a JAK2 inhibitor with a series of serine threonine kinase inhibitors, targeting nine signalling pathways and already used in clinical trials, synergized in inhibiting growth of haematopoietic cells expressing mutant and wild-type forms of JAK2 (V617F) or thrombopoietin receptor (W515L). Out of 15 kinase inhibitors, the ZSTK474 phosphatydylinositol-3′-kinase (PI3K) inhibitor molecule showed strong synergic inhibition by Chou and Talalay analysis with JAK2 and JAK2/JAK1 inhibitors. Other pan-class I, but not gamma or delta specific PI3K inhibitors, also synergized with JAK2 inhibitors. Synergy was not observed in Bcr-Abl transformed cells. The best JAK2/JAK1 and PI3K inhibitor combination pair (ruxolitinib and GDC0941) reduces spleen weight in nude mice inoculated with Ba/F3 cells expressing TpoR and JAK2 V617F. It also exerted strong inhibitory effects on erythropoietin-independent erythroid colonies from MPN patients and JAK2 V617F knock-in mice, where at certain doses, a preferential inhibition of JAK2 V617F mutated progenitors was detected. Our data support the use of a combination of JAK2 and pan-class I PI3K inhibitors in the treatment of MPNs.

## Introduction

A unique somatic acquired Janus kinase 2 (JAK2) tyrosine kinase mutation at position V617F of the pseudokinase domain is associated with the majority of BCR-ABL-negative MPNs 1–4. More than 95% of polycythemia vera (PV), and up to 50% of essential thrombocythemia (ET) and primary myelofibrosis (PMF) patients harbour the JAK2 V617F mutation 5–6. Activating mutations in one of the JAK2-utilizing receptors in the myeloid lineage, the thrombopoietin receptor (TpoR W515L or TpoR W515K), account for about 5–8% of the JAK2 V617F-negative ET and PMF cases 7,8. Both JAK2 and TpoR mutations lead to constitutive activation of JAK2 signalling 7,9. These discoveries indicated JAK2 as a major target for MPN treatment.

Taken together PV, ET and PMF amount in the United States to a prevalence of ∼0.8/1000 11–12, which is significantly higher than chronic myeloid leukaemia. Despite the sizable patient population, no Food and Drug Administration (FDA)-approved drug existed until 2012 for MPNs. As of 2009, several JAK2 inhibitors entered active clinical trials for MPNs 13. In 2012, one JAK2/JAK1 inhibitor, ruxolitinib, was approved by FDA for the treatment of myelofibrosis, following two phase III clinical trials 14–15.

The key cytokine receptors coupled to JAK2 in the myeloid lineage, such as erythropoietin receptor (EpoR) and TpoR (c-MPL), 6–16 are known to trigger activation of phosphatidylinositol-3′-kinase (PI3K) 17,18. JAK2 V617F was shown to activate PI3K, which is required for transformation 1–20. The biology of the cytokine receptor-JAK/signal transducers and activators of transcription (STAT) pathway activation is complicated by interactions with other signalling pathways, themselves also promoting PI3K activation. The receptor tyrosine kinase/RAS/mitogen-activated protein kinase (RTK/RAS/MAPK) pathway was found to interact with the JAK/STAT pathway at multiple levels 21–22. JAKs may phosphorylate insulin receptor substrate (IRS) and p85, which results in the activation of the PI3K pathway 23–24. Input integration from many signalling pathways needs to be considered for treatment of constitutive JAK2 activation-related neoplasms.

All JAK2 inhibitors currently in development are type I competitive ATP-pocket binders/inhibitors of the JH1 kinase domain. The JAK2 V617F mutation occurs in the pseudokinase domain located away from the kinase domain ATP-binding pocket. As such, they are not expected to be specific for JAK2 mutant. JAK2 inhibitors used in the clinics reduce spleen size and diminish constitutional symptoms 14–15. However, the inhibitors are not able to rapidly reverse marrow fibrosis, decrease allele burden or peripheral blasts in myelofibrosis 14–15. These and other challenges facing JAK inhibitor treatment in myelofibrosis 25 could be explained either by insufficient dosing because of anaemia and thrombocytopenia resulting from inhibition of wild-type (WT) JAK2, by addiction to one pathway downstream of JAK2 that needs very low levels of residual JAK activation, by continuous signalling from other JAKs in heteromeric complexes with the inhibited JAK2 26, or by other genetic events that might drive MPN in addition from JAK pathway. On the other hand, recent data obtained after long-term observation (24–48 months) do show that long-term therapy with ruxolitinib retarded advancement of marrow fibrosis, and worsening of marrow fibrosis was significantly more prevalent in the best available therapy arm 27. Evidence has also been reported for a survival advantage of myelofibrosis patients after treatment with ruxolitinib, such as the 3-year data of the COMFORT-II trial 28 or the 2-year data of the COMFORT-I trial 29.

We postulate that MPNs can be more efficiently treated if we target the constitutive JAK2 signalling with a combination of a JAK2 inhibitor with another kinase inhibitor targeting a pathway downstream of the aberrant JAK2 signalling. This should increase targeting specificity, reduce the required doses, and minimize potential side effects of the drugs. To test this hypothesis, we have constructed cell line models of MPN haematopoietic progenitors. These cells are used in a cellular screen by using a JAK2 inhibitor in combination with a panel of kinase inhibitors to identify synergic pairs, which will then be tested in primary cells and explored further by using *in vivo* systems.

## Materials and methods

### Cell lines

Mouse pro-B Ba/F3 cells were first transduced with green fluorescent protein (GFP)-containing bicistronic viruses coding for human WT JAK2 or human JAK2 V617F (cloned into pMX-IRES-GFP) or Bcr-Abl (cloned into MSCV-IRES-GFP) as described previously 10. Populations of cells expressing GFP were isolated by fluorescence-activated cell sorting. Cells stably expressing human JAK2 or JAK2 V617F were subsequently infected with pMX-IRES-GFP retroviruses coding for human WT TpoR, while parental cells were transduced with human TpoR W515L mutant. TpoR was engineered to contain an amino-terminal haemagglutinin (HA) tag 30. Infected cells were sorted for equal HA cell surface expression.

Ba/F3 cells stably expressing TpoR JAK2 WT or JAK2 WT are interleukin-3 (IL3)-dependent for proliferation. IL3 (R&D Systems, Minneapolis, MN, USA) is used at 0.01 μg/ml. Ba/F3 cells expressing JAK2 V617F, TpoR-JAK2 V617F, TpoR W515L or Bcr-Abl are IL3-independent, proliferate to similar extents and exhibit similar levels of STAT5 activation, as measured by luciferase assays with STAT5-dependent luciferase reporters 31 and anti-phospho-Y694 STAT5 western blotting 32. Activation of signalling proteins was determined by Western blot with phospho-specific antibodies, as described 9.

### Drug compounds

**Figure 1:**
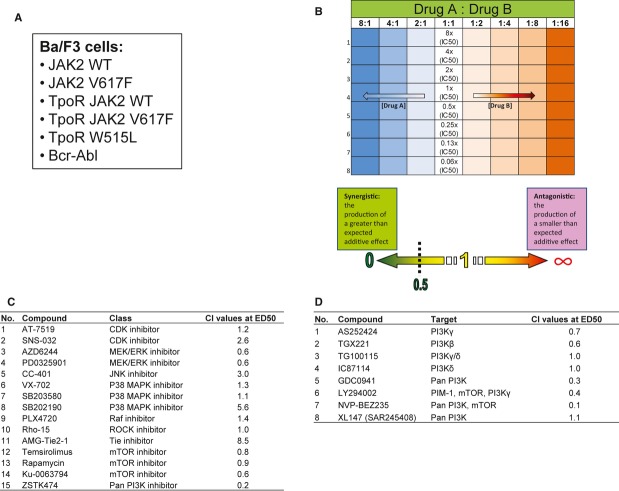
Cell lines and small molecules used in combination for detection of synergy with JAK2 inhibitors in inhibiting proliferation of model myeloproliferative neoplasm cells. (A) Ba/F3 cell lines used for inhibitor screens. Ba/F3 parental and Ba/F3 TpoR JAK2 wild-type (WT) cells were maintained in medium supplemented with IL3, while Ba/F3 JAK2 V617F, TpoR V617F, TpoR W515L and Bcr-Abl were maintained in medium without cytokines. (B) A schematic representation of an 8 × 8 constant ratio design for combination treatment. The two combination drugs were used at their equipotent concentration ratio (IC_50_ of drug A to IC_50_ of drug B is 1:1) in the centre column. The concentration ratio of drug A to drug B is progressively increased towards the left, while the concentration ratio of drug B to drug A is progressively increased towards the right. Each column in the design is a dose–response curve with constant concentration ratio between drug A and drug B. Although usually C.I. <0.8 is considered significant, we selected for combinations showing C.I. <0.5. (C) Combination study using JAK2/JAK1 inhibitor ruxolitinib with various kinase inhibitors at equipotent concentration ratio on TpoR JAK2 V617F cells. (D) Combination study using JAK2/JAK1 inhibitor ruxolitinib with several other PI3K inhibitors at equipotent concentration ratio on TpoR JAK2 V617F cells.

The JAK2/JAK1 inhibitor ruxolitinib (also known as INC424 or INCB018424) (Albany Molecular Research Inc., Albany, NY, USA) and the JAK2 inhibitor TG101348 (SYNthesis Med Chem, San Diego, CA, USA) were used. All compounds were dissolved in 100% dimethyl sulfoxide (Sigma-Aldrich, St. Louis, MO, USA) to prepare 20 mM stocks except for NVP-BEZ235, which was dissolved to prepare 10 mM stock. The identity of compounds used in this study is shown in Figure 1. All compounds were synthesized by SynMedChem except AZD6244 and XL147 (Selleck Chemicals, Houstan, TX, USA), Rapamycin and Temsirolimus (Tocris Bioscience, Bristol, UK), LY294002 from Sigma-Aldrich and SB1518 and CC401 from AMRI (Albany Molecular Research Inc.).

### Design of an 8 × 8 drug combination study and cell viability assay

Combination studies were performed as described 33. Constant ratio combination was used where the two combination drugs were used at their equipotent ratio (*i.e*. at the ratio of their IC_50_s which was pre-determined individually from dose–response studies). An 8 × 8 Latin Square design was constructed with eight different constant molar ratios between the two drugs. Each column was a serial dilution of the two drugs so that eight dose–response curves could be plotted for combination index (CI) calculations (Fig. 1B). The experiments were carried out in quadruplicate in a 384-well plate (Greiner Bio-One, Frickenhausen, Germany) for each pair of drug combination. Volumes of 25 μl of compound mixture and 25 μl of cells (5,000 cells/well) were added to each well. IL3 (0.01 μg/ml) was added to the TpoR JAK2 WT and JAK2 WT cells. Cells were incubated at 37°C for 18 h and then lysed, and ATP levels were measured with a luciferase assay kit (Cell Titer-Glo, Promega, Madison, WI, USA).

### Drug synergism calculation

Combination index was obtained by using the CalcuSyn software version 2.1 (BioSoft, Ferguson, MO, USA). CI values of 0.9–1.1 are considered additive, values of 0.8 and below show moderate-to-strong synergism, while values above 1.2 are moderate-to-strong antagonism for the drug pair tested 34. CIs were calculated only when the goodness of fit of the dose–response curves to the median-effect plot has a correlation coefficient of *r* ≥ 0.9 for the cell-based assays.

### The JAK2 mutant Ba/F3 tumour mouse model

1 × 10^7^ Ba/F3 TpoR JAK2 V617F cells were inoculated intravenously into 7- to 10-week-old female nude mice *via* tail vein injection. Mice were randomly divided into 5–10 per group. Two protocols were used, namely, progression of tumour (leukaemia) burden in mice inoculated with Ba/F3 TpoR JAK2 V617F cells (Fig. S1, Protocol 1); and effect of JAK2 and PI3K inhibitions on reduction in spleen weight (Fig. S1, Protocol 2).

A Vet ABC Hematology Analyzer (Scil, Gurnee, IL, USA) was used for blood counting. Spleen and liver were weighed. Percentages of GFP-positive cells in marrow and peripheral blood mononuclear cells were determined by flow cytometry.

### Colony assays (CFU-E and BFU-E) using bone marrow from JAK2 V617F knock-in and littermate JAK2 wild-type mice

Colony assays (CFU-E and BFU-E) were performed on bone marrow from heterozygous JAK2 V617F or littermate JAK2 WT mice or from mice reconstituted with haematopoietic marrow cells from JAK2 V617F knock-in mice 35, after lethal irradiation, as previously described 32.

1.5–2 × 10^5^ cells were plated in cytokine-depleted methylcellulose medium (M3234) supplemented or not with the indicated cytokines (10 U/ml Epo alone or 5 ng/ml SCF+3 ng/ml IL3 with or without 3 U/ml Epo). Day+2 Epo-independent CFU-E formation was assessed in the presence of ruxolitinib or GDC0941 alone or in combination at several concentrations (0.1–5 μM for ruxolitinib and 1–10 μM for GDC0941) or with vehicle control.

To investigate preference of the combination for JAK2 V617F mutated progenitors, nucleated bone marrow JAK2 V617F and JAK2 WT cells were mixed in a 1:1 ratio (7.5 × 10^4^ cells each) and plated on M3234 methylcellulose medium complemented or not with 3 U/ml Epo, 5 ng/ml SCF and 3 ng/ml IL3. Individual BFU-E colonies were harvested at day 6 and collected in 5 μL water before colony denaturation at 95°C during 10 min. Samples were then genotyped for JAK2 V617F and actin by PCR with the following primers: PR-V617F-for TGT CTT ACT AAA GCC CAG GTG ATG G, PR-V617F-rev GCT CCA GGG TTA CAC GAG TC. PR-actin-for, GGC TGT ATT CCC CTC CAT CG, PR-actin-rev TGG GGG TAC TTC AGG GTC AGG.

### Colony-forming assays on primary human cells

Peripheral blood from two PV patients (JAK2 V617F allele burdens of 49% and 57%) was used to perform BFU-E and CFU-E colony assay. PBMC were collected and plated on methylcellulose medium (H4236 from Stem Cell Technologies, Vancouver, BC, Canada) supplemented or not with 10 U/ml Epo (Eprex: Erythropoietin (Epoetin alfa), Janssen-Cilag, Beerse, Belgium) in triplicate (500,000 cells/plate). Colony formation was assessed at days 7 and 16 for CFU-E and BFU-E colonies, respectively, in presence of drugs alone (0.1 μM ruxolitinib or 1 μM GDC0941), in combination or with vehicle as control.

## Results

### Strong synergy between JAK and PI3K inhibitors in cell-based assays

**Figure 2:**
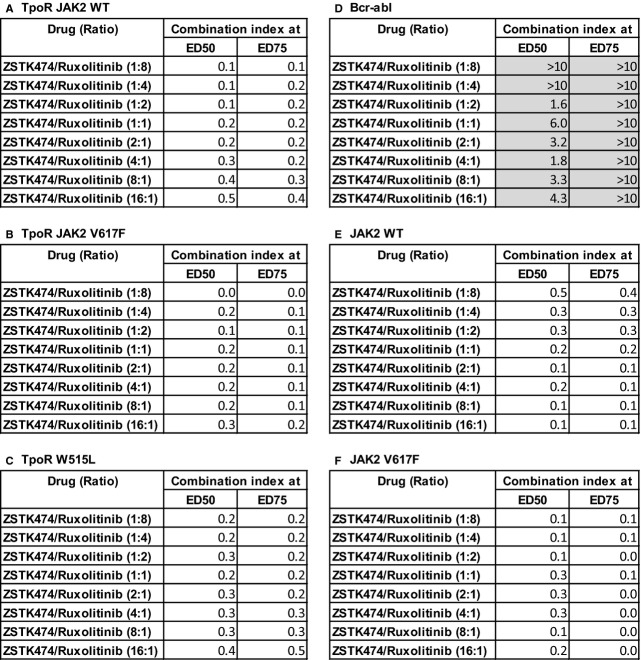
Combination study using JAK2/JAK1 inhibitor ruxolitinib with ZSTK474 in the Ba/F3 cell model. (A) Ba/F3 TpoR JAK2 wild-type (WT) cells; (B) Ba/F3 TpoR JAK2 V617F cells; (C) Ba/F3 TpoR W515L cells; (D) Ba/F3 Bcr-Abl cells; (D and E) Ba/F3 JAK2 WT cells; (F) Ba/F3 JAK2 V617F cells.

Cell lines used in the synergy study are shown in Figure 1A. A summary of CI values (Fig. 1B) for ruxolitinib with the various kinase inhibitors targeting nine signalling pathways in Ba/F3 cells expressing TpoR JAK2 V617F is shown in Figure 1C. Both JAK inhibitors ruxolitinib and TG101348 showed strong drug synergism with PI3K inhibitor ZSTK474 (CI ≤0.5 in more than 50% of the entries in the 8 × 8 Latin Square) in inhibiting the growth of all cell lines, except Bcr-Abl cells, which indicates specificity for cytokine receptor/JAK2 pathway (Fig. 2 for results with ruxolitinib, not shown for results with TG101348). The effective dose ratio ranges for TG101348-ZSTK474 combination are narrower than those observed with the ruxolitinib combination.

**Figure 3:**
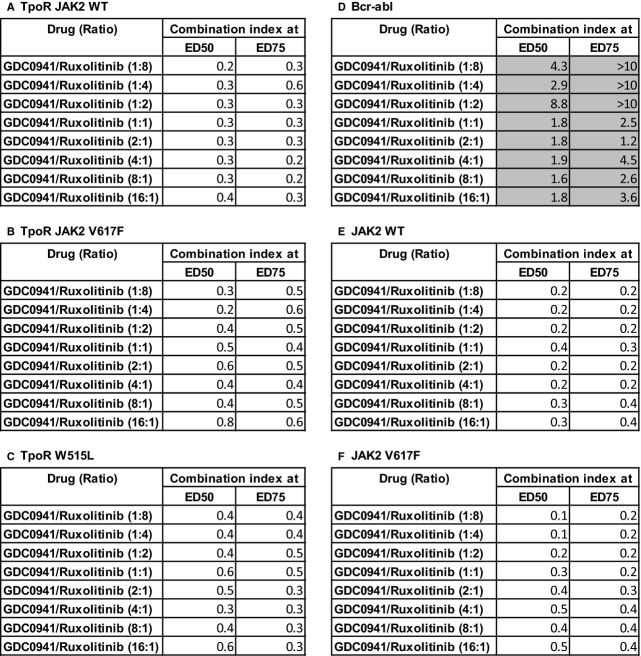
Combination study using the JAK2/JAK1 inhibitor ruxolitinib with GDC0941in the Ba/F3 cell model. Results shown are for eight dose ratios and at two effective doses (ED50 and ED75). (A) Ba/F3 TpoR JAK2 wild-type (WT) cells; (B) Ba/F3 TpoR JAK2 V617F cells; (C) Ba/F3 TpoR W515L cells; (D) Ba/F3 Bcr-Abl cells; (D and E) Ba/F3 JAK2 WT cells; (F) Ba/F3 JAK2 V617F cells.

Subsequently, other PI3K inhibitors were analysed in the combination study. A summary of CI values for ruxolitinib with the PI3K inhibitors in Ba/F3 cells expressing TpoR JAK2 V617F is shown in Figure 1D. Three PI3K inhibitors, NVP-BEZ235, GDC0941 and TGX221 were also found to strongly synergize (CI ≤0.5 in more than 50% of the entries in the 8 × 8 Latin Square) with ruxolitinib or TG101348 (Fig. 3 for results of GDC0941 with ruxolitinib, Fig. S2 for selected PI3K inhibitors with ruxolitinib, results for PI3K inhibitors with TG101348 are not shown). Other tested PI3K inhibitors, especially those specific for the gamma or delta PI3K, were not found to synergize well with the JAK inhibitors.

The synergy between JAK and PI3K inhibitors occurs best when the concentration ratio set based on individual IC50s is in favour of JAK inhibitor over the PI3K inhibitor. This observation implies that the PI3K signalling is secondary to the JAK2 signalling in our cell models. Inhibition of the JAK2 signalling is crucial to sensitize cells to the PI3K inhibitors for specific targeting of the JAK2 or TpoR mutant cells.

Downstream of PI3K is the mTOR pathway, which is considered a major mediator of PI3K signalling. We next tested if the compound Ku-0063794, a specific mTOR inhibitor, will synergize with the JAK1/JAK2 inhibitor ruxolitinib. Ku-0063794 and its derivative, AZD8055, are currently in Phase I/II trial for advance solid tumours, lymphoma and endometrial carcinoma 36. Ku-0063794 strongly synergized with ruxolitinib only in cells stably expressing JAK2 V617F, but not also TpoR (Fig. S3). It remains unclear why JAK2 signalling through TpoR interaction is not targeted effectively by the JAK2-mTOR inhibitors combination, and requires a PI3K inhibitor (Fig. 1C). Nevertheless, a recent study reported that another mTOR inhibitor, everolimus, as a single agent, was effective in a phase 1/2 study of patients with myelofibrosis, supporting the notion that deregulated signalling *via* this pathway is pathogenic in MPNs 37.

### Target specificity of the JAK and PI3K inhibitors

The IC_50_ for cell viability for both JAK inhibitors, ruxolitinib and TG101348, was in the single digit micro molar range. Compared with the JAK2 V617F and TpoR W515L cells, IC_50_ for cell viability was higher with Bcr-Abl transfected cells, but not with JAK2 WT cells (Table 1). The IC values we obtain for Ba/F3 JAK2 V617F cells are comparable to those previously reported 38. We have also observed inhibition of phosphorylation in STAT3, STAT5 and p44/42 (ERK1/2) with ruxolitinib treatment for both WT and mutant JAK2 transfected cells (Fig. S4). This confirms that JAK2 inhibitors are not able to distinguish between WT and mutant JAK2.

**Table 1:** The 50% inhibitory concentrations with their corresponding 95% confidence intervals in parenthesis of the JAK2 and PI3K inhibitors

		IC50 (μM) (95% Confidence Interval)							
		TpoR JAK2 WT	TpoR JAK2 V617F	TpoR W515L	Bcr-abl	JAK2 TW	JAK2 V617F
JAK2 inhibitors	INC424	4.2 (2.4–7.2)	1.6 (1.1–2.4)	0.1 (0.07–0.13)	45.3 (29.9–68.7)	1.0 (0.6–1.7)	0.4 (0.2–0.6)
	TG101348	1.4 (1.3–1.5)	0.8 (0.7–0.9)	0.8 (0.7–1.0)	2.7 (2.2–3.3)	1.8 (1.5–2.3)	0.6 (0.6–0.7)
PI3K inhibitors	ZSTK474	225.2 (170.7–297.2)	80.5 (56.9–113.9)	64.3 (47.1–87.8)	125.9 (76.7–206.4)	250.5 (173.6–361.6)	116.5 (99.7–136.2)
	NVP-BEZ235	36.8 (23.9–56.6)	3.5 (2.7–4.6)	1.8 (1.4–2.5)	>200	7.5 (4.5–12.7)	0.04 (0.02–0.06)
	GDC0941	192.3 (127.6–289.7)	17.9 (13.8–23.1)	43.7 (28.3–67.6)	147.2 (61.5–352.2)	185.1 (165.6–206.8)	25.8 (10.7–61.8)
	TGX221	76.1 (67.4–86.0)	49.4 (44.7–54.5)	50.0 (45.9–54.6)	75.9 (66.3–86.9)	28.3 (15.1–53.2)	20.39 (11.9–34.9)

On the other hand, the engineered Ba/F3 cells were not very responsive to PI3K inhibitors alone, in the absence of JAK2 inhibitors. IC_50_ for cell viability was in the double to triple digit micro molar range with NVP-BEZ235 being more potent than other PI3K inhibitors tested (Table 1), possibly because this compound targets both PI3K and mTOR. TGX221 is a class I PI3Kβ-specific inhibitor, whereas ZSTK474, GDC0941 and NVP-BEZ235 are pan-class I PI3K inhibitors. It is unclear at present why the pan PI3K inhibitors have a wide range of IC_50_ values and why only certain pan PI3K inhibitors demonstrated synergism with the JAK2 inhibitors in inhibiting Ba/F3 cell growth. All four PI3K inhibitors seemed to be selective for cells expressing JAK2 V617F and TpoR W515L, while cells expressing WT JAK2 or Bcr-Abl were more resistant towards the PI3K inhibitors (Fig. S2).

### Effect of inhibitors on signalling pathways

**Figure 4:**
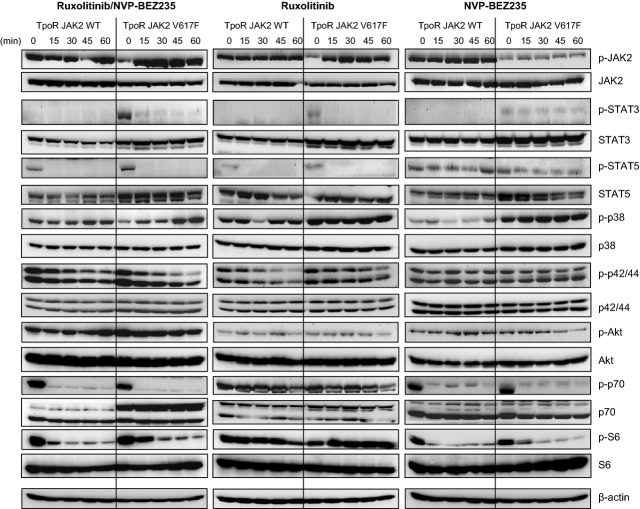
Effect of the JAK2/JAK1 inhibitor ruxolitinib and PI3K inhibitor NVP-BEZ236 alone or in combination on signalling in Ba/F3 TpoR cells overexpressing JAK2 wild-type (WT) or JAK2 V617F. Cells expressing TpoR JAK2 V617F were autonomous for growth, while Ba/F3 TpoR JAK2 WT cells were grown in medium supplemented with IL3. Cells were treated for the indicated intervals with 1 μM ruxolitinib and 10 μM NVP-BEZ235 alone or in combination and western blotting was performed with the phospho-specific antibodies (phospho-JAK2 (Tyr1007/1008), phospho-STAT3 (Tyr705), phospho-STAT5 (Tyr694), phospho-p70 S6 kinase (Thr389), phospho-S6 ribosomal protein (Ser235/236), phospho-AKT (Ser473), phospho-p38 MAP-kinase (Thr180/Tyr182), phospho-p44/42 ERK1/2 (Thr202/Tyr204), with antibodies to actin (as loading control) and with antibodies detecting the signalling proteins themselves.

We examined the effect of JAK2 and PI3K inhibitors alone and in combination on our model Ba/F3 cell lines. As expected, phosphorylation at Y1007 of JAK2 was stabilized by ruxolitinib because of the type I mechanism of inhibition, where molecules bind to active state kinases, block catalytic activity, while maintaining open conformation of activation loop continues to be phosphorylated by other kinases 39. We observed inhibition by NVP-BEZ235 of p70 S6 kinase and S6 ribosomal protein phosphorylation (both are downstream effectors of PI3K signalling) when JAK2 or JAK2 V617F cells were co-expressed with TpoR (Fig. 4) or not (Fig. S4A). The effect of NVP-BEZ235 was less pronounced in the cell line expressing the active mutant TpoR W515L (Fig. S4B). Ruxolitinib inhibited the STAT activation (STAT5 and STAT3), as expected. In contrast, ruxolitinib did not inhibit STAT5 and STAT3 phosphorylation in Ba/F3 cells expressing Bcr-Abl (Fig. S4C). Interestingly, NVP-BEZ235 weakly inhibited STAT5 activation in TpoR expressing cells, but not in JAK2 or JAK2 V617F cells (Fig. 4, Fig. S4A and B).

In combination, JAK2 and PI3K inhibitors blocked both STAT and downstream PI3K targets p70 S6 kinase and S6 ribosomal protein (Fig. 4 and Fig. S4). Akt phosphorylation at S473 (mTORC2 site) was only marginally inhibited by NVP-BEZ235 in TpoR cells, and was not inhibited in JAK2 or JAK2 V617F cells, with almost no effect by the combination. The combination appears to weakly diminish activation of ERK1/2, but not of p38 MAP-kinase (Fig. 4, Fig. S4A). Overall, strong effects were seen with the drug combination on the JAK/STAT and certain PI3K downstream pathways (p70 S6 kinase). These data suggest that the synergic inhibition is because of the PI3K- p70 S6 kinase arm and less so because of inhibition of the PI3K-Akt arm. Interestingly, an Akt inhibitor, MK-2206 was shown also to synergize with ruxolitinib in cell lines, MPN primary cells and a retroviral reconstitution model of TpoR W515L mutant 40. In aggregate, inhibition of both arms of the PI3K pathway may prove very effective in MPNs, along with JAK2.

### Effect of JAK2 and PI3K inhibitors on spleen weight in the Ba/F3 TpoR JAK2 V617F mouse model

**Figure 5:**
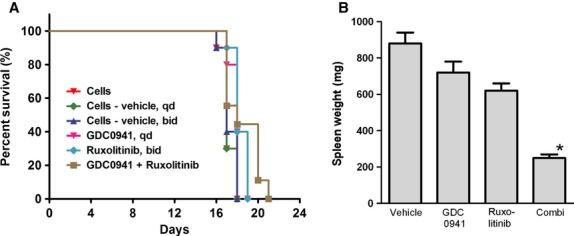
Effect of JAK and PI3K inhibitions on survival (A) and spleen weight (B) in the JAK2 mutation-driven leukaemia mouse model. Ba/F3 TpoR JAK2 V617F cells (1 × 10^7^) were intravenously inoculated into 8- to 10-week-old female nude mice. After 3 days, mice were treated with either vehicle, GDC0941 alone at 50 mg/kg body weight, ruxolitinib alone at 50 mg/kg body weight, or a combination of both compounds for 12 days. Combination treatment increased survival by 2 days compared with single compound treatment. Combination treatment also reduced spleen weight significantly compared with vehicle-treated mice, or mice treated with GDC0941 alone, or ruxolitinib alone (*P < 0.001 for each comparison). qd: every day, bid: twice a day.

Female BALB/c nude mice injected with Ba/F3 TpoR JAK2 V617F cells were studied for overall survival and enlargement of spleen (Fig. 5). Tissue samples were collected from mice killed on day 8 and day 14. Spleen weight increased after 8 days of cell implantation, while increase of liver weight was observed by day 14 (Fig. S5A). A progressive increase of Ba/F3 TpoR JAK2 V617F cells in the systemic blood circulation and spleen was observed from day 8 to day 14 (Fig. S5B). An increase of Ba/F3 TpoR JAK2 V617F cells in the bone marrow was observed on day 8 (results not shown). Platelet and red blood cell numbers decreased when compared with the control groups, most likely because of replacement by proliferating Ba/F3 transformed cells (Fig. S5C).

The mice inoculated with Ba/F3 TpoR JAK2 V617F cells were either treated with vehicle alone, GDC0941 alone, ruxolitinib alone or a combination of GDC0941 and ruxolitinib (Fig. S1, see Protocol 2). An evaluation of *in vivo* drug–drug interactions between ruxolitinib and GDC0941 is documented in Table S1. GDC0941 was chosen over NVP-BEZ235 for this study because of solubility issue with NVP-BEZ235 in our oral formulation.

All mice in the treated groups with the compound combination at 50 mg/kg body weight had no overt sign of clinical distress except for enlarged organs. At day 12 post-inoculation, spleen weights of mice treated with the compound combination were ∼70% less than the spleen weights of mice treated with vehicle alone, and ∼60% less than the spleen weights of mice treated with GDC0941 alone or ruxolitinib alone at 50 mg/kg body weight. Combination treatment extended survival when compared with vehicle control and to individual treatments (Fig. 5A). The increase in size and weight of spleen was also significantly delayed (*P* < 0.001) in mice receiving the combination treatment (Fig. 5B).

### Effect of JAK2 and PI3K inhibitors on colony assays from JAK2 V617F knock-in mice and MPN patient cells

**Figure 6:**
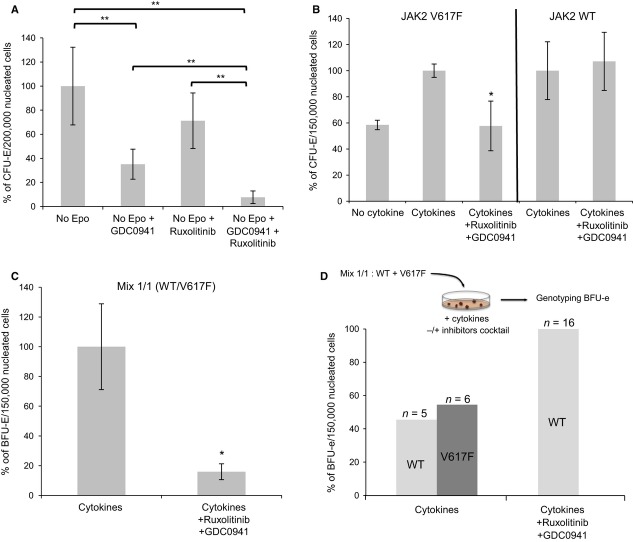
Effect of JAK and PI3K inhibitors on colony formation from heterozygous JAK2 V617F knock-in bone marrow cells and mixtures of knock-in and wild-type (WT) mice. (A) Effect of ruxolitinib (5 μM) and GDC 0942 (10 μM) or combination thereof on Epo-independent CFU-E (2% foetal serum, no cytokines) from bone marrow of reconstituted heterozygous JAK2 V617F knock-in mice. The number of colonies was normalized to those in the untreated controls in the absence of Epo (936 ± 302 EEC/200,000 bone marrow nucleated heterozygous JAK2 V617F knock-in cells). Shown are averages of two mice, each performed in duplicate (average of six points) ±SD. **P < 0.01 unpaired Student's test with unequal variance. (B) Comparison between generation of CFU-E colonies between bone marrow cells from JAK2 V617F knock-in mice and JAK2 WT littermate mice. Cells were plated in methylcellulose in the presence of cytokines (3 U/ml Epo, 5 ng/ml SCF, 3 ng/ml IL3) alone or with the combination of 0.1 μM ruxolitinib and 1 μM GDC 0941. The number of colonies was normalized to those in the untreated controls in presence of cytokines, which was 1099 ± 56/150,000 bone marrow nucleated heterozygous JAK2 V617F cells and 837 ± 185/150,000 bone marrow nucleated WT JAK2 cells. Shown are averages of triplicates ±SD of one representative experiment out of two. *P < 0.05 unpaired Student's test with unequal variance. (C) Bone marrow cells from JAK2 V617F knock-in mice and WT littermate mice were mixed in a 1:1 ratio (75,000 cells each) and BFU-E formation was determined in the absence or presence of the combination of 0.1 μM ruxolitinib and 1 μM GDC 0941 in the presence of cytokines (3 U/ml Epo, 5 ng/ml SCF, 3 ng/ml IL3). The number of colonies was normalized to those in the untreated controls in the presence of cytokines, which was 339 ± 98 BFU-E colonies/150,000 nucleated cells. Shown are averages of triplicates ±SD of one representative experiment out of two. *P < 0.05 unpaired Student's test with unequal variance. (D) Preferential inhibition of JAK2 V617F knock-in BFU-E compared with WT BFU-E by the combination of 0.1 μM ruxolitinib and 1 μM GDC 0941. 1:1 mixed JAK2 V617F knock-in and WT cells were plated in methylcellulose in the presence of cytokines (3 U/ml Epo, 5 ng/ml SCF, 3 ng/ml IL3) alone or with the combination of inhibitors. BFU-E colonies were harvested at day 6 from three plates for each condition and genotyped for JAK2 V617F. Three plates were used for each condition. Genotyping was performed on 4, 2 and 5 BFU-Es for the condition ‘cytokines’ and 4, 6 and 6 for the condition ‘cytokines + inhibitor’ combination. Values represent% of BFU-Es genotyped as JAK2 V617F-positive or JAK2 WT-positive per 150,000 nucleated mixed cells. Shown are the results of one representative experiment out of two.

We performed colony-forming unit (CFU) assays to assess effect of drugs combination on formation of colonies from bone marrow of mice that were knock-in for JAK2 V617F in their haematopoietic system following bone marrow transplantation with JAK2 V617F knock-in bone marrow 32–35. As shown in Figure 6A, CFU-Es were observed at day 2 in the absence of Epo, indicating the capacity of those colonies to growth independently of cytokines (EEC). Both ruxolitinib and GDC0941 used individually were able to inhibit EEC formation at 5 and 10 μM, respectively (Fig. 6A). Furthermore, combination of those drugs at the same concentration drastically blocks EEC formation.

A similar synergy induced by the combination of the two inhibitors could be detected on patient derived day 7 Epo-independent CFU-E (Fig. S6A) and day 16 BFU-E (Fig. S6B).

We aimed to assess whether this synergic effect showed any preference for JAK2 V617F colonies, especially at concentrations of ruxolitinib inhibitor that are lower than those utilized in the clinic, given their known toxicities. We chose (lower) concentrations that fit with the IC50 data obtained from our Ba/F3 TpoR JAK2 V617F cells for the combination (Table S1), or to the IC50 data obtained for individual ruxolitinib treatment of the Ba/F3 JAK2 V617F and Ba/F3 TpoR W515L cells (Table 1), namely 0.1 μM ruxolitinib and 1 μM GDC0941 and 0.3 μM ruxolitinib and 3 μM GDC0941. These concentrations are also fitting well with pharmacokinetics measurements in mice injected with 50 mg/kg body ruxolitinib and with a recent study that examined synergy between an Akt inhibitor and ruxolitinib 40. We then tested whether there was any preference for inhibition of JAK2 V617F *versus* JAK2 WT CFU-Es by the ruxolitinib and GDC0941 combination.

To this end, we performed two types of experiments. In the first, we compared the effects of the combination on CFU-E formation from JAK2 V617F heterozygous knock-in and littermate JAK2 WT bone marrow cells. In Figure 6B, we show that there is a statistically significant preference for inhibiting the JAK2 V617F CFU-Es. In the second type of experiments, we mixed 1:1 WT and JAK2 V617F knock-in bone marrow cells and BFU-Es were counted in two conditions, with cytokines and with cytokines and combination of inhibitors (Fig. 6C), and then individual colonies were picked and genotyped for JAK2 V617F and reference actin (Fig. 6D). Results showed a preference for inhibition of the mutated colonies. In one such experiment, of 11 colonies derived from three independent plates not treated with inhibitor, six BFU-Es were JAK2 V617F, while in the presence of the inhibitor combination, all 16 colonies were negative for JAK2 V617F. These results show that preference of the combination for the JAK2 V617F colonies can be observed.

## Discussion

The Chou-Talalay method of determining CI values has rigorous mathematical proof and extensive validation 41 and is more accurate than descriptive drug combination studies 42,43. Progressively smaller CI values are an estimation of progressively stronger synergism. The accepted cut-off value for synergism is ≤0.8 34. Here, we used a cut-off value of ≤0.5 to ensure that only very strong synergistic drug-pairs are selected.

TGX221 (PI3Kβ inhibitor) is in research phase, while ZSTK474, NVP-BEZ235 and GDC0941 (all pan PI3K inhibitors) are in clinical trials. A comprehensive study 45 showed that ZSTK474 and GDC0941 have highly similar inhibition profiles and specificity for class I PI3K, while NVP-BEZ235 is different, being able to inhibit both PI3K and mTOR. Other isoform specific PI3K inhibitors (against γ and δ isoforms) analysed in this study were not found to synergize with the JAK2 inhibitors. Our findings support earlier studies that PI3Kα and PI3Kβ isoforms play an important role in tumourigenesis 46–47, while PI3Kδ and PI3Kγ are involved in other diseases 48–49. Furthermore, the NVP-BEZ235 was found by Fiskus *et al*. to synergize with JAK2 inhibition in inhibiting growth of cultured and primary MPN cells 50. In our study, we show that these effects can be detected in primary JAK2 V617F knock-in and patient cells, and in an *in vivo* tumourigenic system based on JAK2 V617F expression. Our finding is that cells expressing constitutively active JAK2 mutants are dependent on the PI3K pathway for their survival. Possible dose-reduction of ruxolitinib by combined use of a pan type I PI3K inhibitor may result in the reduction of adverse events of ruxolitinib, and this may represent a major benefit of the combination therapy.

Although MEK/ERK/p38 and JNK pathways are activated downstream of cytokine receptors 5–6, none of the MAP-kinase inhibitors strongly synergized (CI <0.5) with JAK2 inhibitors in our assays. However, the two MEK/ERK dual inhibitors that we tested, AZD6244 and PD0325901 did synergize with a CI of 0.6 (Fig. 1C), which qualifies as a weak synergy by the Chou-Talalay method, suggesting that the MEK/ERK pathway does contribute to the cytokine-independent growth of MPN cells. In previous studies, we have shown that severe MPN and myelofibrosis are induced by TpoR W515A mutant *via* the cytosolic TpoR tyrosine (Y626) that connects to the shc-ras-MEK-ERK1/2 pathway 9, and that the same tyrosine residue was required for down-modulation of TpoR in JAK2 V617F by excessive ERK1/2 and STAT3 activation, again linked to severe MPN 32. Finally, the ERK1/2 pathway is involved in the antiproliferative and senescence effects induced by Tpo *via* TpoR in cells that physiologically express high JAK2 levels, such as late megakaryocytes 32–51. Altogether excessive MAP-kinase ERK1/2 and STAT3 activation might be linked to myelofibrosis, and contribute to cytokine-independent growth, while the PI3K appears to be the major pathway linked to proliferation. These results strengthen the existing evidence of molecular crosstalk between the JAK/STAT and the PI3K pathways in various tissues 52,53 and supported the importance of a combination treatment targeting both JAK2 and the PI3K signalling. Mechanistically, it has been shown that activated mutant forms of STAT5 interact with the regulatory subunit p85 of PI3K, *via* the adaptor Gab2 55. Further studies will be required to assess the precise mechanism by which inhibiting PI3K synergizes with JAK2 in blocking MPN cell proliferation. Importantly, we could observe the synergic action of JAK2 and PI3K inhibitors also in cells cultivated in defined medium in the absence of serum, which indicates that PI3K activation is largely derived from JAK2 activation.

Our results showed that Ba/F3 TpoR JAK2 V617F cells can propagate *in vivo* in nude mice leading to accumulation of GFP-positive cells and anaemia, thrombocytopenia, splenomegaly and hepatomegaly. This was also reported with Ba/F3 JAK2 V617F cells 56. Splenomegaly was most prominent during early development of this oncogenic model with spleen weight as a good indicator of disease progression. We demonstrated that simultaneous inhibition of JAK2 and PI3K signalling pathways led to significantly delayed splenomegaly in mice inoculated with Ba/F3 TpoR JAK2 V617F cells.

Furthermore, combining JAK2 and PI3K inhibitors inhibited Epo-independent CFU-E and BFU-E colony formation from primary cells from JAK2 V617F-positive MPN patients and JAK2 V617F knock-in mice. The Ruxolitinib-GDC0941 combination, used at certain doses, exerted preferential inhibitory effects on JAK2 V617F knock-in CFU-E and BFU-E. These data suggest that careful dosing can achieve specificity for the mutated progenitors.

Our pre-clinical studies indicate that the ruxolitinib and GDC0941 combination is safe and effective when administered orally. Clinical trials with JAK2/JAK1 or JAK2 inhibitors indicate that these drugs are uniformly active based on reduction in patient spleen size and improvement in constitutional symptoms, but they induce varying toxicity profiles 15–59. Our findings suggest that we may be able to administer these drugs at lower concentrations when adding PI3K inhibitors. Our study gives the first evidence of synergistic effects of JAK2 inhibitors with PI3K inhibitors and provides a framework for combination trials using compounds in these two classes in patients with MPNs.

## Supporting Information

**Figure S1.** Protocols for in vivo tumour model of Ba/F3 TpoR JAK2 V617F cells in nude mice. **Figure S2.** Combination study with ruxolitinib and PI3K inhibitors. **Figure S3.** Combination study with ruxolitinib and Ku0063794 (mTOR inhibitor). **Figure S4.** Effects of JAK and PI3K inhibitors on signalling via the JAK2, STAT3/STAT5, PI3K, mTOR and MAPK/ERK1/2 in the Ba/F3 JAK2 WT and BaF3 JAK2 V617F (A), Ba/F3 TpoR wt and TpoR-W515L (B) or Ba/F3 Bcr-Abl (C) cell lines. **Figure S5.** Effects of inoculating Ba/F3 TpoR JAK2 V617F cells into BALB/c mice. **Figure S6.** Effects of the combination of JAK and PI3K inhibitorson Epo-independent CFU-Es (EECs) and Epo-independent BFU-Es from PV patients. **Table S1.** Pharmacokinetic profile of ruxolitinib and GDC0941 when administered in combination to nude mice injected with Ba/F3 TpoR JAK2 V617F cells.
