# Supplementary material for: Combination treatment for myeloproliferative neoplasms using JAK and pan-class I PI3K inhibitors
Source: J Cell Mol Med. 2013 Nov 19;17(11):1397–409. doi: 10.1111/jcmm.12156 (PMC4117552; doi:10.1111/jcmm.12156)
Supplement: Supplementary file 1 — Figure S1. Protocols for in vivo tumour model of Ba/F3 TpoR JAK2 V617F cells in nude mice. Figure S2. Combination study with ruxolitinib and PI3K inhibitors. Figure S3. Combination study with ruxolitinib and Ku0063794 (mTOR inhibitor). Figure S4. Effects of JAK and PI3K inhibitors on signalling via the JAK2, STAT3/STAT5, PI3K, mTOR and MAPK/ERK1/2 in the Ba/F3 JAK2 WT and BaF3 JAK2 V617F (A), Ba/F3 TpoR wt and TpoR-W515L (B) or Ba/F3 Bcr-Abl (C) cell lines. Figure S5. Effects of inoculating Ba/F3 TpoR JAK2 V617F cells into BALB/c mice. Figure S6. Effects of the combination of JAK and PI3K inhibitorson Epo-independent CFU-Es (EECs) and Epo-independent BFU-Es from PV patients. Table S1. Pharmacokinetic profile of ruxolitinib and GDC0941 when administered in combination to nude mice injected with Ba/F3 TpoR JAK2 V617F cells. [file jcmm0017-1397-sd1.pdf]

Supplemental Figure 1. Protocols for *in vivo* tumor model of Ba/F3 TpoR JAK2 V617F cells in nude mice.

A. Supplementary Protocol 1: Progression of leukemia in mice inoculated with Ba/F3 TpoR JAK2 V617F GFP cells.

**Groups**	**N**	**Inoculation**
Day 8 - c	5	Ba/F3 TpoR JAK2 V617F GFP cells
Day 8 - m	3	Medium
Day 8 - b	3	-
Day 14 - c	5	Ba/F3 TpoR JAK2 V617F GFP cells
Day 14 - m	3	Medium
Day 14 - b	3	-

Mice in group Day 8 - c and group Day 14 - c (n = 5 per group) were inoculated with 1×10^7 of Ba/F3 TpoR JAK2 V617F GFP cells via tail vein injection. Mice in group Day 8 – m and group Day 14 – m (n = 3 per group) were inoculated with 100 µl of cell culture medium without any cells. Mice in group Day 8 – b and group Day 14 – b (n = 3 per group) received neither cells nor medium; they are called blank. Mice were euthanized on day 8 and day 14 using CO_2 gas. Spleen, liver and blood samples were collected. N is the number of mice per group.

B. Supplementary Protocol 2: Effect of JAK2 and PI3K inhibitions on mice spleen weight

**Groups**	**N**	**Agent**	**Dose (mg/kg)**	**Route**	**Schedule**
Day 16 - V	5	Vehicle	200 µl/20 g; 100 µl/20 g	po	bid
Day 16 - 50G	5	GDC0941	50	po	qd
Day 16 - 50I	5	Ruxolitinib	50; 50	po	bid
Day 16 - 50GI	5	GDC0941 + Ruxolitinib	50+50; 50	po	qd; bid

Ba/F3 TpoR JAK2 V617F GFP cells (1×10^7) were intravenously inoculated into 8 – 10 weeks old female nude mice. The inoculated mice were randomly organized into 5 per group (N). After 3 days, mice were treated with either vehicle, GDC0941 alone, ruxolitinib alone or the combination of GDC0941 and ruxolitinib. Mice in group Day 16 – V were orally administered twice a day (bid) with vehicle at 200 μl/20 g in the morning and 100 μl/20 g in the evening (8 hours difference from the morning dose). The vehicle comprised of a mixture of 0.5% methylcellulose and 0.1% Tween-80. Mice in group Day 16 – 50G were orally administered once a day (qd) with GDC0941 alone at 50 mg/kg. The volume of administration of GDC0941 was 200 μl/20 g. Mice in group Day 16 – 50I were orally administered twice a day (bid) with ruxolitinib alone at 50 mg/kg body weight. Mice in group Day 16 - 50GI were orally administered twice a day (bid) with the combination of 50 mg/kg GDC0941 and ruxolitinib in the morning, and with ruxolitinib alone 50 mg/kg in the evening. The volume of drug administration for Day 16 – 50I group and Day 16 – 50GI group followed that of Day 16 – V group. After 12 days of treatment, mice were euthanized using CO_2 gas. Spleens were collected. qd: every day, bid: twice a day, po: per oral.

Supplemental Figure 2. Combination study with ruxolitinib and PI3K inhibitors. The combination indices for 3 effective doses (ED) are shown for Ruxolitinib with ZSTK474 (A), NVP-BEZ235 (B), GDC0941 (C) and TGX221 (D). ED50 represents effective dose resulting in 50% cell viability. Boxes shaded in grey have combination index values larger than 0.8.

A.

TpoR JAK2 WT with IL3

Drug (Ratio)	Combination Index at		
	ED50	ED75	ED90
ZSTK474/Ruxolitinib (1:8)	0.1	0.1	0.2
ZSTK474/Ruxolitinib (1:4)	0.1	0.2	0.2
ZSTK474/Ruxolitinib (1:2)	0.1	0.2	0.2
ZSTK474/Ruxolitinib (1:1)	0.2	0.2	0.3
ZSTK474/Ruxolitinib (2:1)	0.2	0.2	0.2
ZSTK474/Ruxolitinib (4:1)	0.3	0.2	0.3
ZSTK474/Ruxolitinib (8:1)	0.4	0.3	0.3
ZSTK474/Ruxolitinib (16:1	0.5	0.4	0.3

Bcr-abl

Drug (Ratio)	Combination Index at		
	ED50	ED75	ED90
ZSTK474/Ruxolitinib (1:8)	>10	>10	>10
ZSTK474/Ruxolitinib (1:4)	>10	>10	>10
ZSTK474/Ruxolitinib (1:2)	1.6	>10	>10
ZSTK474/Ruxolitinib (1:1)	6.0	>10	>10
ZSTK474/Ruxolitinib (2:1)	3.2	>10	>10
ZSTK474/Ruxolitinib (4:1)	1.8	>10	>10
ZSTK474/Ruxolitinib (8:1)	3.3	>10	>10
ZSTK474/Ruxolitinib (16:1	4.3	>10	>10

TpoR JAK2 V617F

Drug (Ratio)	Combination Index at		
	ED50	ED75	ED90
ZSTK474/Ruxolitinib (1:8)	0.0	0.0	0.1
ZSTK474/Ruxolitinib (1:4)	0.2	0.1	0.1
ZSTK474/Ruxolitinib (1:2)	0.1	0.1	0.1
ZSTK474/Ruxolitinib (1:1)	0.2	0.1	0.1
ZSTK474/Ruxolitinib (2:1)	0.2	0.1	0.1
ZSTK474/Ruxolitinib (4:1)	0.2	0.1	0.1
ZSTK474/Ruxolitinib (8:1)	0.2	0.1	0.1
ZSTK474/Ruxolitinib (16:1	0.3	0.2	0.1

JAK2 WT with IL3

Drug (Ratio)	Combination Index at		
	ED50	ED75	ED90
ZSTK474/Ruxolitinib (1:8)	0.5	0.4	0.3
ZSTK474/Ruxolitinib (1:4)	0.3	0.3	0.3
ZSTK474/Ruxolitinib (1:2)	0.3	0.3	0.2
ZSTK474/Ruxolitinib (1:1)	0.2	0.2	0.2
ZSTK474/Ruxolitinib (2:1)	0.1	0.1	0.2
ZSTK474/Ruxolitinib (4:1)	0.2	0.1	0.1
ZSTK474/Ruxolitinib (8:1)	0.1	0.1	0.2
ZSTK474/Ruxolitinib (16:1	0.1	0.1	0.1

TpoR W515L

Drug (Ratio)	Combination Index at		
	ED50	ED75	ED90
ZSTK474/Ruxolitinib (1:8)	0.2	0.2	0.2
ZSTK474/Ruxolitinib (1:4)	0.2	0.2	0.2
ZSTK474/Ruxolitinib (1:2)	0.3	0.2	0.2
ZSTK474/Ruxolitinib (1:1)	0.2	0.2	0.2
ZSTK474/Ruxolitinib (2:1)	0.3	0.2	0.2
ZSTK474/Ruxolitinib (4:1)	0.3	0.3	0.2
ZSTK474/Ruxolitinib (8:1)	0.3	0.3	0.3
ZSTK474/Ruxolitinib (16:1	0.4	0.5	0.6

JAK2 V617F

Drug (Ratio)	Combination Index at		
	ED50	ED75	ED90
ZSTK474/Ruxolitinib (1:8)	0.1	0.1	0.2
ZSTK474/Ruxolitinib (1:4)	0.1	0.1	0.0
ZSTK474/Ruxolitinib (1:2)	0.1	0.0	0.0
ZSTK474/Ruxolitinib (1:1)	0.3	0.1	0.0
ZSTK474/Ruxolitinib (2:1)	0.3	0.0	0.0
ZSTK474/Ruxolitinib (4:1)	0.3	0.0	0.0
ZSTK474/Ruxolitinib (8:1)	0.1	0.0	0.0
ZSTK474/Ruxolitinib (16:1	0.2	0.0	0.0

B.

TpoR JAK2 WT with IL3

Drug (Ratio)	Combination Index at		
	ED50	ED75	ED90
GDC0941/Ruxolitinib (1:8)	0.2	0.3	0.4
GDC0941/Ruxolitinib (1:4)	0.3	0.6	1.1
GDC0941/Ruxolitinib (1:2)	0.3	0.3	0.4
GDC0941/Ruxolitinib (1:1)	0.3	0.3	0.3
GDC0941/Ruxolitinib (2:1)	0.3	0.3	0.3
GDC0941/Ruxolitinib (4:1)	0.3	0.2	0.2
GDC0941/Ruxolitinib (8:1)	0.3	0.2	0.2
GDC0941/Ruxolitinib (16:1	0.4	0.3	0.2

Bcr-abl

Drug (Ratio)	Combination Index at		
	ED50	ED75	ED90
GDC0941/Ruxolitinib (1:8)	4.3	>10	>10
GDC0941/Ruxolitinib (1:4)	2.9	>10	>10
GDC0941/Ruxolitinib (1:2)	8.8	>10	>10
GDC0941/Ruxolitinib (1:1)	1.8	2.5	9.8
GDC0941/Ruxolitinib (2:1)	1.8	1.2	2.1
GDC0941/Ruxolitinib (4:1)	1.9	4.5	>10
GDC0941/Ruxolitinib (8:1)	1.6	2.6	7.3
GDC0941/Ruxolitinib (16:1	1.8	3.6	>10

TpoR JAK2 V617F

Drug (Ratio)	Combination Index at		
	ED50	ED75	ED90
GDC0941/Ruxolitinib (1:8)	0.3	0.5	0.6
GDC0941/Ruxolitinib (1:4)	0.2	0.6	1.6
GDC0941/Ruxolitinib (1:2)	0.4	0.5	0.7
GDC0941/Ruxolitinib (1:1)	0.5	0.4	0.4
GDC0941/Ruxolitinib (2:1)	0.6	0.5	0.5
GDC0941/Ruxolitinib (4:1)	0.4	0.4	0.5
GDC0941/Ruxolitinib (8:1)	0.4	0.5	0.7
GDC0941/Ruxolitinib (16:1	0.8	0.6	0.5

JAK2 WT with IL3

Drug (Ratio)	Combination Index at		
	ED50	ED75	ED90
GDC0941/Ruxolitinib (1:8)	0.2	0.2	0.2
GDC0941/Ruxolitinib (1:4)	0.2	0.2	0.3
GDC0941/Ruxolitinib (1:2)	0.2	0.2	0.3
GDC0941/Ruxolitinib (1:1)	0.4	0.3	0.2
GDC0941/Ruxolitinib (2:1)	0.2	0.2	0.2
GDC0941/Ruxolitinib (4:1)	0.2	0.2	0.3
GDC0941/Ruxolitinib (8:1)	0.3	0.4	0.4
GDC0941/Ruxolitinib (16:1	0.3	0.4	0.5

TpoR W515L

Drug (Ratio)	Combination Index at		
	ED50	ED75	ED90
GDC0941/Ruxolitinib (1:8)	0.4	0.4	0.4
GDC0941/Ruxolitinib (1:4)	0.4	0.4	0.4
GDC0941/Ruxolitinib (1:2)	0.4	0.5	0.6
GDC0941/Ruxolitinib (1:1)	0.6	0.5	0.4
GDC0941/Ruxolitinib (2:1)	0.5	0.3	0.3
GDC0941/Ruxolitinib (4:1)	0.3	0.3	0.3
GDC0941/Ruxolitinib (8:1)	0.4	0.3	0.2
GDC0941/Ruxolitinib (16:1	0.6	0.3	0.2

JAK2 V617F

Drug (Ratio)	Combination Index at		
	ED50	ED75	ED90
GDC0941/Ruxolitinib (1:8)	0.1	0.2	0.4
GDC0941/Ruxolitinib (1:4)	0.1	0.2	0.2
GDC0941/Ruxolitinib (1:2)	0.2	0.2	0.2
GDC0941/Ruxolitinib (1:1)	0.3	0.2	0.3
GDC0941/Ruxolitinib (2:1)	0.4	0.3	0.3
GDC0941/Ruxolitinib (4:1)	0.5	0.4	0.3
GDC0941/Ruxolitinib (8:1)	0.4	0.4	0.3
GDC0941/Ruxolitinib (16:1	0.5	0.4	0.4

C.

TpoR JAK2 WT with IL3

Drug (Ratio)	Combination Index at		
	ED50	ED75	ED90
BEZ235/Ruxolitinib (1:8)	0.7	0.4	0.2
BEZ235/Ruxolitinib (1:4)	0.8	0.3	0.1
BEZ235/Ruxolitinib (1:2)	0.6	0.3	0.1
BEZ235/Ruxolitinib (1:1)	0.6	0.2	0.1
BEZ235/Ruxolitinib (2:1)	0.5	0.2	0.1
BEZ235/Ruxolitinib (4:1)	0.4	0.3	0.2
BEZ235/Ruxolitinib (8:1)	0.3	0.3	0.3
BEZ235/Ruxolitinib (16:1)	2.1	9.4	>10

Bcr-abl

Cells not responsive to treatment

TpoR JAK2 V617F

Drug (Ratio)	Combination Index at		
	ED50	ED75	ED90
BEZ235/Ruxolitinib (1:8)	0.3	0.1	0.1
BEZ235/Ruxolitinib (1:4)	0.2	0.1	0.0
BEZ235/Ruxolitinib (1:2)	0.2	0.1	0.0
BEZ235/Ruxolitinib (1:1)	0.2	0.1	0.1
BEZ235/Ruxolitinib (2:1)	0.2	0.1	0.1
BEZ235/Ruxolitinib (4:1)	0.2	0.2	0.2
BEZ235/Ruxolitinib (8:1)	0.2	0.3	0.4
BEZ235/Ruxolitinib (16:1)	1.2	0.8	0.6

JAK2 WT with IL3

Drug (Ratio)	Combination Index at		
	ED50	ED75	ED90
BEZ235/Ruxolitinib (1:8)	0.0	0.0	0.0
BEZ235/Ruxolitinib (1:4)	0.0	0.0	0.0
BEZ235/Ruxolitinib (1:2)	0.0	0.0	0.0
BEZ235/Ruxolitinib (1:1)	0.0	0.0	0.0
BEZ235/Ruxolitinib (2:1)	0.0	0.0	0.0
BEZ235/Ruxolitinib (4:1)	0.0	0.0	0.0
BEZ235/Ruxolitinib (8:1)	0.1	0.1	0.3
BEZ235/Ruxolitinib (16:1)	0.2	0.2	0.7

TpoR W515L

Drug (Ratio)	Combination Index at		
	ED50	ED75	ED90
BEZ235/Ruxolitinib (1:8)	0.0	0.0	0.0
BEZ235/Ruxolitinib (1:4)	0.1	0.1	0.2
BEZ235/Ruxolitinib (1:2)	0.3	0.2	0.1
BEZ235/Ruxolitinib (1:1)	0.4	0.2	0.1
BEZ235/Ruxolitinib (2:1)	1.3	0.4	0.1
BEZ235/Ruxolitinib (4:1)	3.0	0.7	0.2
BEZ235/Ruxolitinib (8:1)	2.0	0.6	0.2
BEZ235/Ruxolitinib (16:1)	2.2	0.9	0.4

JAK2 V617F

Drug (Ratio)	Combination Index at		
	ED50	ED75	ED90
BEZ235/Ruxolitinib (1:8)	0.0	0.0	0.1
BEZ235/Ruxolitinib (1:4)	0.0	0.1	0.1
BEZ235/Ruxolitinib (1:2)	0.1	0.0	0.1
BEZ235/Ruxolitinib (1:1)	0.1	0.0	0.0
BEZ235/Ruxolitinib (2:1)	0.2	0.0	0.1
BEZ235/Ruxolitinib (4:1)	0.2	0.1	0.2
BEZ235/Ruxolitinib (8:1)	0.2	0.1	0.3
BEZ235/Ruxolitinib (16:1)	0.3	0.1	0.5

D.

TpoR JAK2 WT with IL3

Drug (Ratio)	Combination index at		
	ED50	ED75	ED90
TGX221/Ruxolitinib (1:8)	0.8	0.7	0.5
TGX221/Ruxolitinib (1:4)	0.9	0.6	0.4
TGX221/Ruxolitinib (1:2)	0.7	0.4	0.2
TGX221/Ruxolitinib (1:1)	0.6	0.3	0.1
TGX221/Ruxolitinib (2:1)	0.6	0.2	0.1
TGX221/Ruxolitinib (4:1)	0.5	0.2	0.1
TGX221/Ruxolitinib (8:1)	0.5	0.2	0.1
TGX221/Ruxolitinib (16:1	0.7	0.2	0.1

Bcr-abl

Drug (Ratio)	Combination Index at		
	ED50	ED75	ED90
TGX221/Ruxolitinib (1:8)	0.6	0.2	0.1
TGX221/Ruxolitinib (1:4)	0.4	0.1	0.0
TGX221/Ruxolitinib (1:2)	0.4	0.1	0.0
TGX221/Ruxolitinib (1:1)	0.8	0.3	0.2
TGX221/Ruxolitinib (2:1)	0.8	0.2	0.1
TGX221/Ruxolitinib (4:1)	1.2	0.5	0.3
TGX221/Ruxolitinib (8:1)	0.6	0.1	0.0
TGX221/Ruxolitinib (16:1	2.7	4.3	7.3

TpoR JAK2 V617F

Drug (Ratio)	Combination index at		
	ED50	ED75	ED90
TGX221/Ruxolitinib (1:8)	0.4	0.5	0.9
TGX221/Ruxolitinib (1:4)	0.6	0.5	0.5
TGX221/Ruxolitinib (1:2)	0.8	0.4	0.3
TGX221/Ruxolitinib (1:1)	0.9	0.7	0.6
TGX221/Ruxolitinib (2:1)	0.8	0.5	0.5
TGX221/Ruxolitinib (4:1)	0.8	0.9	1.2
TGX221/Ruxolitinib (8:1)	0.8	0.7	0.6
TGX221/Ruxolitinib (16:1	1.0	1.0	1.1

JAK2 WT with IL3

Drug (Ratio)	Combination index at		
	ED50	ED75	ED90
TGX221/Ruxolitinib (1:8)	0.6	0.5	0.5
TGX221/Ruxolitinib (1:4)	0.7	0.6	0.5
TGX221/Ruxolitinib (1:2)	0.8	0.5	0.3
TGX221/Ruxolitinib (1:1)	0.8	0.4	0.2
TGX221/Ruxolitinib (2:1)	0.6	0.3	0.2
TGX221/Ruxolitinib (4:1)	0.5	0.3	0.2
TGX221/Ruxolitinib (8:1)	0.3	0.3	0.2
TGX221/Ruxolitinib (16:1	0.7	0.4	0.2

TpoR W515L

Drug (Ratio)	Combination index at		
	ED50	ED75	ED90
TGX221/Ruxolitinib (1:8)	0.4	0.7	1.1
TGX221/Ruxolitinib (1:4)	1.1	1.0	0.9
TGX221/Ruxolitinib (1:2)	1.2	0.8	0.6
TGX221/Ruxolitinib (1:1)	1.2	0.7	0.4
TGX221/Ruxolitinib (2:1)	0.9	0.5	0.3
TGX221/Ruxolitinib (4:1)	1.0	0.5	0.3
TGX221/Ruxolitinib (8:1)	0.7	0.4	0.2
TGX221/Ruxolitinib (16:1	0.7	0.4	0.2

JAK2 V617F

Drug (Ratio)	Combination index at		
	ED50	ED75	ED90
TGX221/Ruxolitinib (1:8)	0.6	0.6	0.6
TGX221/Ruxolitinib (1:4)	0.7	0.6	0.6
TGX221/Ruxolitinib (1:2)	0.7	0.5	0.4
TGX221/Ruxolitinib (1:1)	0.9	0.6	0.4
TGX221/Ruxolitinib (2:1)	0.8	0.6	0.5
TGX221/Ruxolitinib (4:1)	0.7	0.6	0.5
TGX221/Ruxolitinib (8:1)	0.5	0.5	0.5
TGX221/Ruxolitinib (16:1	1.0	0.8	0.6

Supplemental Figure 3. Combination study with ruxolitinib and Ku0063794 (mTOR inhibitor). Boxes shaded in grey have combination index values larger than 0.8. ED50 represents effective dose resulting in 50% cell viability.

TpoR JAK2 WT with IL3

Drug (Ratio)	Combination index at		
	ED50	ED75	ED90
Ku0063794/Ruxolitinib (1:8)	0.6	0.9	1.2
Ku0063794/Ruxolitinib (1:4)	0.7	1.2	2.2
Ku0063794/Ruxolitinib (1:2)	0.7	0.9	1.3
Ku0063794/Ruxolitinib (1:1)	0.7	0.6	0.7
Ku0063794/Ruxolitinib (2:1)	0.7	0.5	0.6
Ku0063794/Ruxolitinib (4:1)	0.9	0.6	0.8
Ku0063794/Ruxolitinib (8:1)	1.4	0.9	1.1
Ku0063794/Ruxolitinib (16:1	3.3	6.6	>10

Bcr-abl

Unable to generate dose response curves

TpoR JAK2 V617F

Drug (Ratio)	Combination index at		
	ED50	ED75	ED90
Ku0063794/Ruxolitinib (1:8)	0.2	1.1	5.3
Ku0063794/Ruxolitinib (1:4)	0.5	1.2	2.9
Ku0063794/Ruxolitinib (1:2)	0.8	1.0	1.4
Ku0063794/Ruxolitinib (1:1)	0.6	0.5	0.7
Ku0063794/Ruxolitinib (2:1)	1.0	0.5	0.4
Ku0063794/Ruxolitinib (4:1)	1.2	0.5	0.4
Ku0063794/Ruxolitinib (8:1)	1.9	0.5	0.2
Ku0063794/Ruxolitinib (16:1	2.6	0.7	0.3

JAK2 WT with IL3

Drug (Ratio)	Combination index at		
	ED50	ED75	ED90
Ku0063794/Ruxolitinib (1:8)	0.6	1.3	2.9
Ku0063794/Ruxolitinib (1:4)	0.4	1.2	3.0
Ku0063794/Ruxolitinib (1:2)	0.7	0.8	1.0
Ku0063794/Ruxolitinib (1:1)	0.5	0.7	1.0
Ku0063794/Ruxolitinib (2:1)	0.4	0.6	1.0
Ku0063794/Ruxolitinib (4:1)	0.3	0.5	0.9
Ku0063794/Ruxolitinib (8:1)	0.4	0.7	1.7
Ku0063794/Ruxolitinib (16:1	0.8	>10	>10

TpoR W515L

Drug (Ratio)	Combination index at		
	ED50	ED75	ED90
Ku0063794/Ruxolitinib (1:8)	0.5	0.8	1.4
Ku0063794/Ruxolitinib (1:4)	0.5	1.0	2.0
Ku0063794/Ruxolitinib (1:2)	1.0	1.1	1.4
Ku0063794/Ruxolitinib (1:1)	1.9	1.2	0.9
Ku0063794/Ruxolitinib (2:1)	2.1	1.0	0.6
Ku0063794/Ruxolitinib (4:1)	1.8	0.9	0.6
Ku0063794/Ruxolitinib (8:1)	2.7	1.2	0.7
Ku0063794/Ruxolitinib (16:1	3.9	1.4	0.6

JAK2 V617F

Drug (Ratio)	Combination index at		
	ED50	ED75	ED90
Ku0063794/Ruxolitinib (1:8)	0.1	0.2	0.2
Ku0063794/Ruxolitinib (1:4)	0.1	0.1	0.2
Ku0063794/Ruxolitinib (1:2)	0.2	0.2	0.1
Ku0063794/Ruxolitinib (1:1)	0.3	0.2	0.1
Ku0063794/Ruxolitinib (2:1)	0.4	0.2	0.1
Ku0063794/Ruxolitinib (4:1)	0.3	0.1	0.1
Ku0063794/Ruxolitinib (8:1)	0.4	0.2	0.1
Ku0063794/Ruxolitinib (16:1	0.5	0.2	0.1

Supplemental Figure 4. Effects of JAK and PI3K inhibitors on signaling via the JAK2, STAT3/STAT5, PI3K, mTOR and MAPK/ERK1/2 in the Ba/F3 JAK2 WT and BaF3 JAK2 V617F (A), Ba/F3 TpoR wt and TpoR-W515L (B) or Ba/F3 Bcr-Abl (C) cell lines. The cells were treated with 1 μM INCB018424 or 10 μM NVP-BEZ235 or a combination of both kinase inhibitors. Western blotting was performed with the phospho-specific antibodies (phospho-JAK2 (Tyr1007/1008), phospho-STAT3 (Tyr705), phospho-STAT5 (Tyr694), phospho-p70 S6 kinase (Thr389), phospho-S6 ribosomal protein (Ser235/236), phospho-AKT (Ser473), phospho-p38 MAP kinase (Thr180/Tyr182), phospho-p44/42 ERK1/2 (Thr202/Tyr204), with antibodies to actin (as loading control) and with antibodies detecting the signaling proteins themselves.

A.

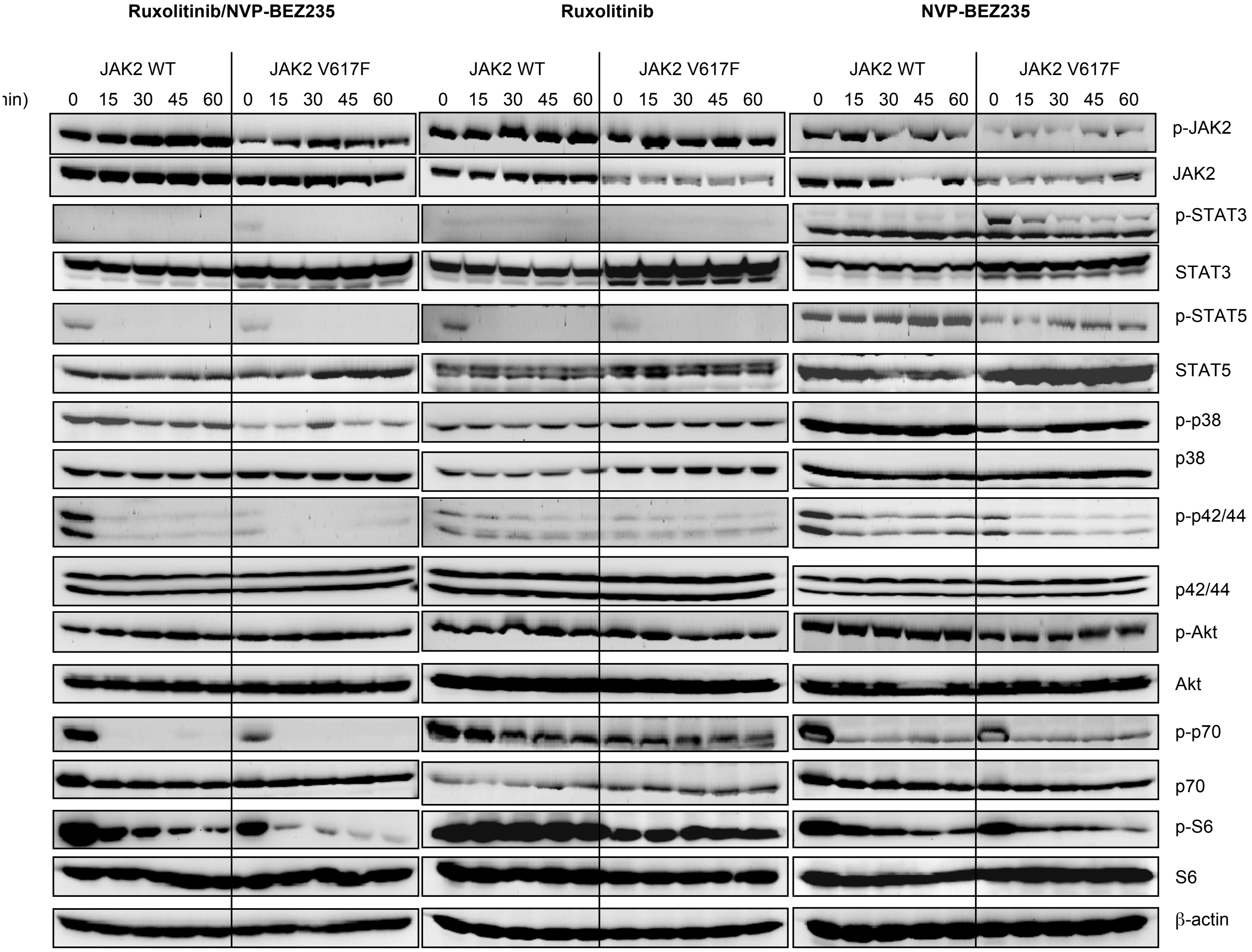

B.

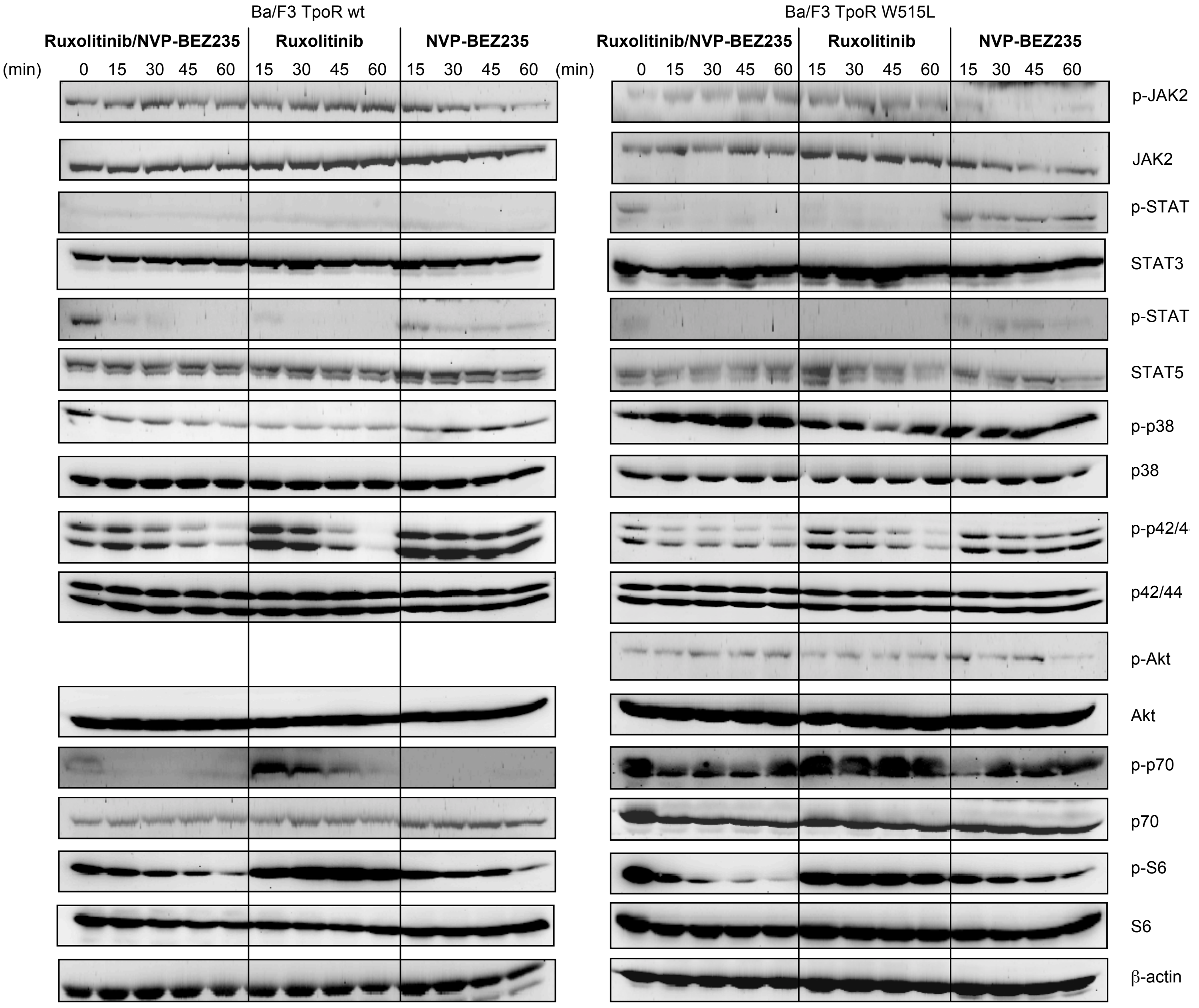

Ba/F3 TpoR wt
Ba/F3 TpoR W515L
Ruxolitinib/NVP-BEZ235
Ruxolitinib
NVP-BEZ235
(min)
0 15 30 45 60 15 30 45 60 15 30 45 60
p-JAK2
JAK2
p-STAT
STAT3
p-STAT
STAT5
p-p38
p38
p-p42/4
p42/44
p-Akt
Akt
p-p70
p70
p-S6
S6
β-actin

C.

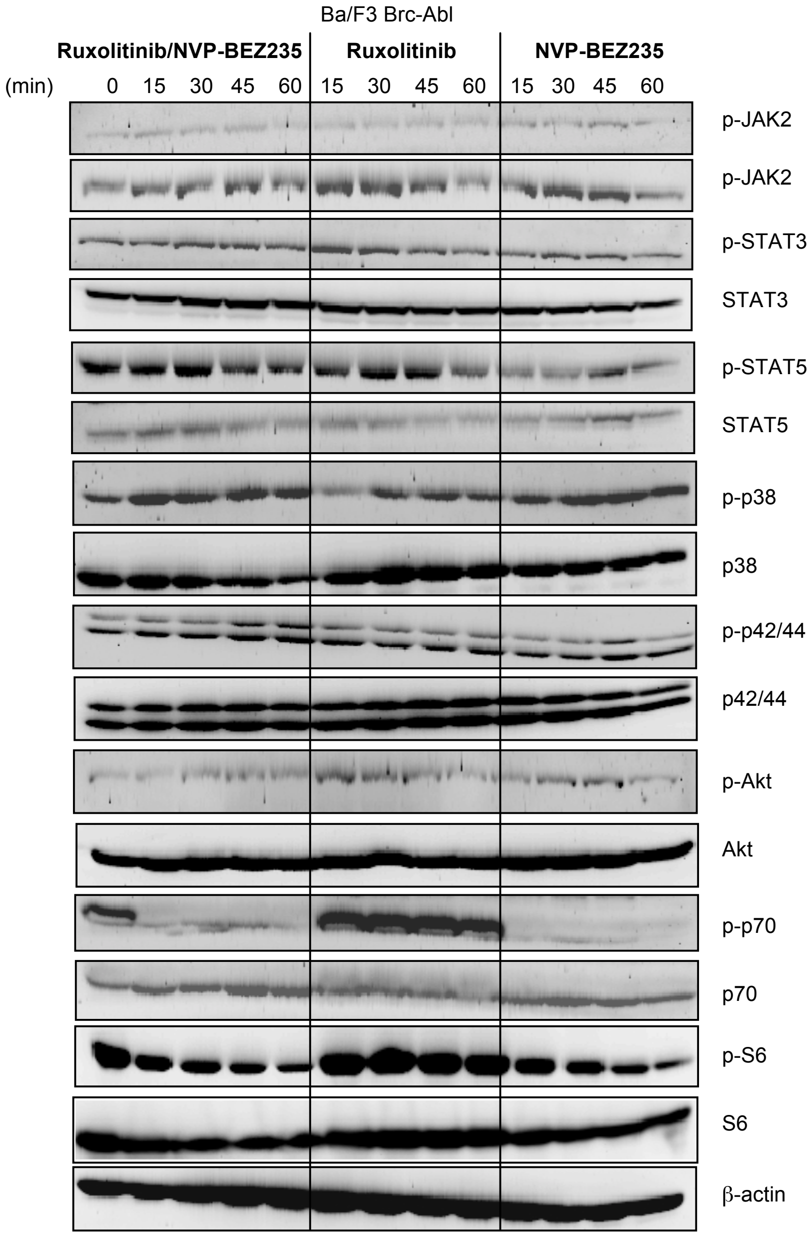
Ba/F3 Brc-Abl
Ruxolitinib/NVP-BEZ235
Ruxolitinib
NVP-BEZ235
(min)
0 15 30 45 60 15 30 45 60 15 30 45 60
p-JAK2
p-JAK2
p-STAT3
STAT3
p-STAT5
STAT5
p-p38
p38
p-p42/44
p42/44
p-Akt
Akt
p-p70
p70
p-S6
S6
β-actin

Supplemental **Figure 5.** Effects of inoculating Ba/F3 TpoR JAK2 V617F cells into BALB/c mice. The cells (1 × 10^7) were intravenously inoculated into 8 – 10 weeks old female nude mice. Organ enlargements were observed after 8 days (A). Proliferation of Ba/F3 TpoR JAK2 V617F cells in different organ compartments of the inoculated mice. By day 14, 40% of PBMC and 20% of spleen cells were Ba/F3 cells (B). Blood profiles of inoculated mice showing increase in WBC, and decrease in both RBC and platelets (C).

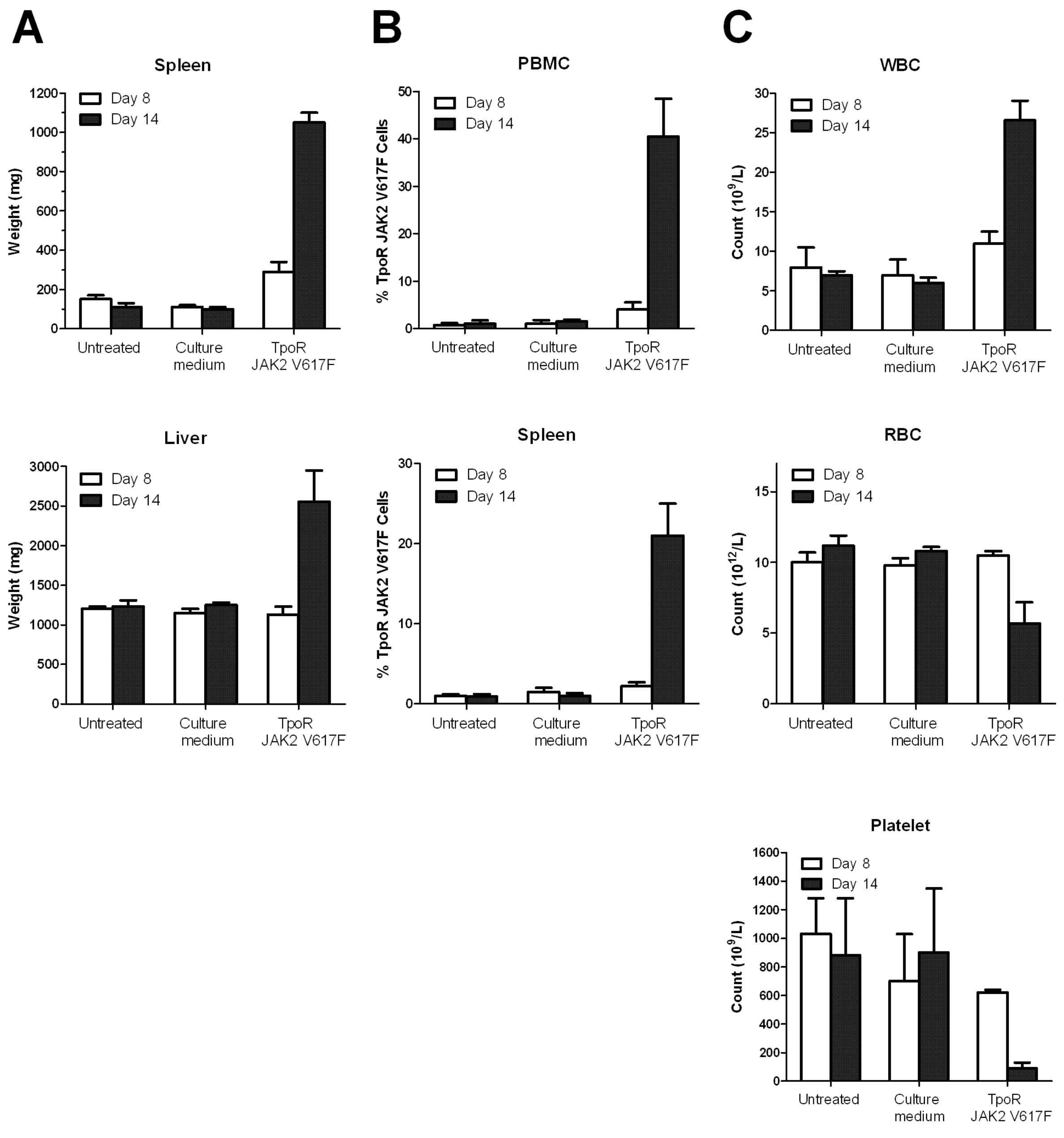

Supplemental Figure 6. Effects of the combination of JAK and PI3K inhibitors on Epo-independent CFU-Es (EECs) and Epo-independent BFU-Es from PV patients.

A. Day 7 EECs derived from a PV patient with 57% JAK2 V617F allele burden. Shown are averages of duplicates.

B. BFU-E colonies were detected at day 16 in presence of drugs alone (0.1 μM ruxolitinib or 1 μM GDC0941) and in combination or with vehicle as control in BFU-E assays where 500,000 peripheral PBMCs of a JAK2 V617F-positive PV patient (allele burden 49%) were plated per well. Shown results are average of triplicates ± SD. Epo was used as positive control at 10 U/ml.

A

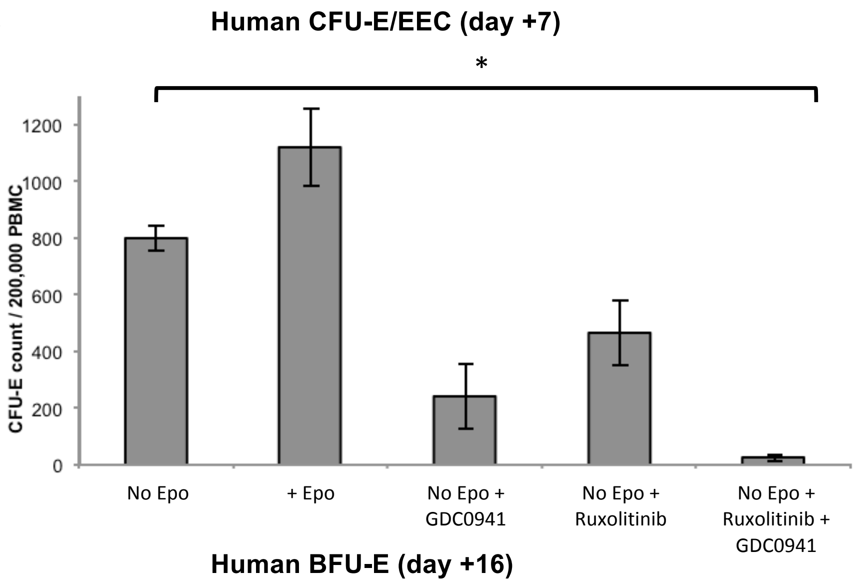

B

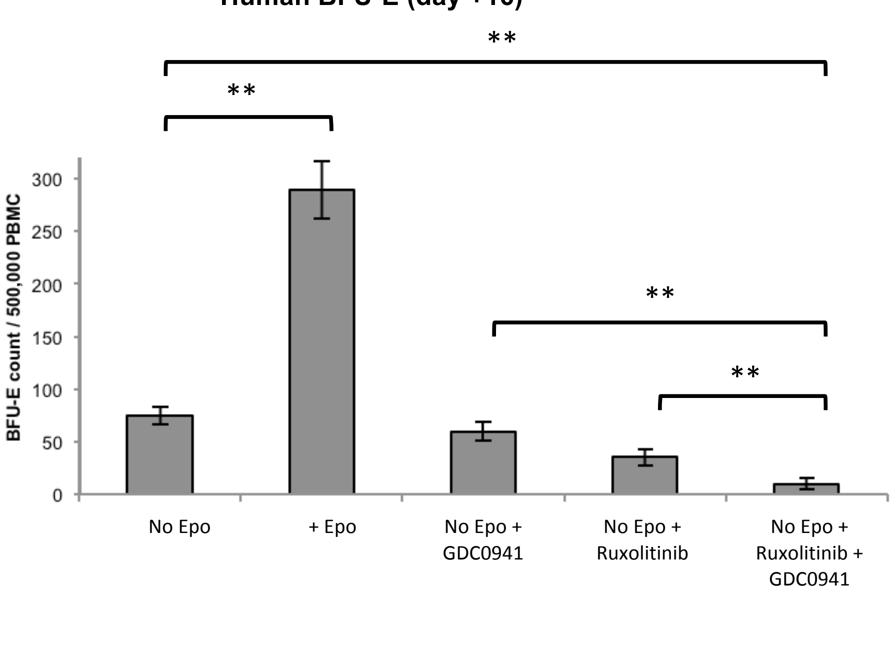

Supplemental Table 1. Pharmacokinetic profile of ruxolitinib and GDC0941 when administered in combination to nude mice injected with Ba/F3 TpoR JAK2 V617F cells. Plasma (n = 3) were harvested for 8 time points within 24 hours of treatment.

**Pharmacokinetics - oral parameters**	**Unit**	**Ruxolitinib**	**Ruxolitinib from combined Ruxolitinib and GDC0941**	**GDC0941**	**GDC0941 from combined Ruxolitinib and GDC0941**
C_{max}	ng/ml	15879	15270	9779	10350
T_{max}	hour	0.17	0.17	0.17	0.5
$T_{1/2}$	hour	0.83	1.48	1.58	1.82
$AUC_{0\text{-}inf}$	ng.h/ml	9135	9855	37912	42716
C_{max}/IC_{50}	-	104	100	1.2	1.3
$AUC_{0\text{-}inf}/IC_{50}$	-	60	64	4.8	5.4

Note: IC_{50} values were 153 ng/ml for ruxolitinib and 7858 ng/ml for GDC0941 in *in vitro* cytotoxicity assay using Ba/F3 TpoR JAK2 V617F cells.
